# Perceived sexual harassment and gender differences in anesthesiology: a cross-sectional survey

**DOI:** 10.3389/fpubh.2025.1478340

**Published:** 2025-02-12

**Authors:** Renuka Shenoy, Ronald Harter, Alberto Uribe, Mahmoud Abdel-Rasoul, Jillian Tishko, Tristan Weaver, Erica Stein

**Affiliations:** ^1^Department of Anesthesiology, The Ohio State University Wexner Medical Center, Columbus, OH, United States; ^2^Center of Biostatistics, The Ohio State University, Columbus, OH, United States

**Keywords:** sexual harassment, #MeToo, #MeTooMedicine, gender equality, anesthesiology

## Abstract

**Background:**

In 2017, the hashtags #MeToo and #MeTooMedicine went viral and raised awareness of sexual harassment (SH) and sexual violence affecting all ages, genders, races, sexualities, and careers. Most studies investigating SH have found that women report higher rates of SH experiences compared to men, with documented incidences reaching as high as 81%. Notably, 47% of these incidents go unreported. A study from the Association of Anesthesiologists found that female victims of sexual assault often do not speak up due to the myth that harassment is rare, which further silences women and stigmatizes this issue, leading them to be discouraged from participating in academic anesthesia. Due to the outdated data examining the incidence of SH in the field of anesthesiology, an anonymous 20 item survey was administered to physician attendings and trainees to assess the incidence of SH perceiving SH within the field.

**Methods:**

An anonymous questionnaire-based cross-sectional study, adapted from a validated survey tool on SH and burnout, was administered to a sample of registered members of the American Society of Anesthesiologists (ASA) to evaluate physicians’ perceptions related to SH and burnout in their workplace.

**Results:**

The email survey was sent to 30,765 registered ASA members; a total of 2,830 (9.2%) members responded to the 20-item survey for this quantitative analysis. 53.4% (*n* = 1,511) and 44.2% (*n* = 1,251) identified themselves as men and women, respectively. Among all the respondents, 57.6% perceived some form of SH (75.5% women and 44.2% men, *p*-value:<0.0001). Around 40.1% of respondents indicated that they did not react to or report SH events, with a significantly higher incidence among women when compared to men (46.3% vs. 35.5%; *p*-value:<0.0001). Respondents who perceived SH were more likely to agree with the 5-point Likert-scale questions related to experiencing burnout, facing declining job opportunities or leaving a job, and becoming more emotionally detached from others.

**Conclusion:**

SH has been reported by both male and female anesthesiologists, with female physicians perceiving significantly higher rates of SH compared to their male counterparts. Most respondents who experienced SH in their workplaces expressed agreement with statements related to “burnout feelings” and/or “declining a job opportunity or leaving the workplace.” This study contributes to the current literature that SH is prevalent within the field of anesthesiology. Furthermore, our study demonstrates that SH has a positive correlation to feelings of burnout. This study demonstrates the critical importance of instituting policies regarding reporting SH events. Additionally, implementing bystander training can empower individuals to report witnessed SH events. Lastly, safeguards should be implemented to protect those who report witnessing or experiencing SH events.

## Introduction

The United States Equal Employment Opportunity Commission defines harassment as imposing unwanted and inappropriate behaviors (1). Sexual harassment (SH) specifically is defined as a harassment involving the requests for sexual favors, offensive comments, or physical sexual conduct (1). Stereotypical gender jokes and quid pro quo sexual exchanges are examples of SH (2). One of the most experienced forms of SH is making inappropriate remarks about one’s body (2).

In the fall of 2017, the hashtag #MeToo went viral. #MeToo raised awareness of SH and sexual violence affecting all ages, genders, races, sexualities, and careers. The hashtag signifies solidarity, but it also highlights the high prevalence of SH in society. Twenty-four hours after actress Alyssa Milano posted #MeToo on social media, Facebook reported 12 million #MeToo related posts and comments (2). This movement initiated the conversation around SH, particularly the impact of SH upon a person’s well-being (1–4).

Since the beginning of the #MeToo movement, the hashtag #MeTooMedicine started trending as well (5). Consequently, multiple studies have been conducted worldwide indicating the prevalence of SH and gender discrimination within the medical field (6–12) and to discuss the importance of recognizing SH in the workplace (2, 13, 14). These studies have been conducted in different medical disciplines, such as emergency medicine, cardiothoracic surgery, orthopedics, among others (6–10). Furthermore, the Association of American Medical Colleges (AAMC) conducted their own report to understand and address SH in academic medicine using data from the 2019–2020 AAMC Faculty Engagement Survey (14). The AAMC 2022 report exposed the prevalence of SH in the medical field, particularly in surgery and anesthesia. This could be due to historical gender power dynamics in the operating room (15). As part of its findings, the AAMC 2022 report revealed that among 27 basic and clinical disciplines, the field of anesthesiology had the highest incidence of women (52.6%) and men (21.3%) experiencing gender-based SH (14).

Overall, most studies show that women reported higher rates of sexual discrimination and SH in comparison to men, with a reported incidence as high as 81% in women (6–10, 16). The reported literature identifies SH as the third most common type of harassment that medical residents face during training (7). Disappointingly, around 47% of these events are not reported due to fear of harming one’s personal reputation along with fear of retribution, dismissal and/or its impact on one’s future career options (9, 10, 16, 17).

After experiencing SH, physicians often encounter thoughts of feeling unsafe, degradation, and self-blame in addition to burnout (18). Burnout is defined as a psychological syndrome of emotional exhaustion, depersonalization and reduced personal accomplishment that can trigger episodes of depression or suicide attempts (13). Burnout after SH experiences have been linked to poor health, alcoholism, depression, and suicidal ideation among physicians (1, 19).

As part of its findings, the AAMC 2022 report revealed that among 27 basic and clinical disciplines, the field of anesthesiology had the highest incidence of women (52.6%) and men (21.3%) experiencing gender-based SH (12). However, there is scarce other data examining the incidence of SH and the types of SH within the field (8). Therefore, we conducted an anonymous questionnaire-based cross-sectional study of a convenience sample of anesthesia care providers with the objective of assessing the prevalence of perceived SH reported by members of the American Society of Anesthesiologists (ASA).

## Methods

### Study design

An anonymous questionnaire-based cross-sectional study, adapted from a validated survey tool on SH and burnout (4, 18, 19), was administered to a sample of registered members of the ASA to evaluate physicians’ perceptions related to SH and burnout in their workplace.

### Study population

After an institutional review Board (IRB) protocol review and approval from our local IRB, Office of Responsible Research Practices (ORRP)—The Ohio State University in 2020 (protocol #2020E0694), active members registered with the ASA were contacted by email requesting to respond an anonymous electronic survey. The survey of physician attendings and trainees in the field of anesthesiology was distributed via email on August 1, 2020, and responses were collected through September 15, 2020. The survey was active for 45 days and a reminder email was sent 2 weeks and 1 week prior to closing the survey’s access. The invitation detailed that the primary aim of the survey was to examine perceived SH within the field of anesthesiology. The survey was administered using an online, secure platform through Qualtrics (Qualtrics, Provo, UT).

### Survey instrument and measurements (Appendix A)

An anonymous questionnaire-based cross-sectional study was adapted from a validated survey tool on SH and burnout (20) and administered to a sample of registered members of the American Society of Anesthesiologists (ASA) to evaluate physicians’ perceptions related to SH and burnout in their workplace. The adapted survey had 20 items and was designed to collect information regarding types of perceived SH experiences in the workplace (gender harassment, unwanted sexual attention and sexual coercion), physician wellness, and burnout. The survey also collected basic demographic data including gender identity, number of years in training and practice, geographic location of practice, and type of anesthesia care provider. In addition, the survey collected data regarding incidences and types of SH, the setting in which the incidences occurred, perpetrator type, and the level of training of those involved at the time of the SH. Questions regarding responses to perceived SH and changes in behavior such as burnout were also included.

SH was characterized into the following three sub-categories: *gender harassment* (being told sexually suggestive stories, having crude sexual remarks directed at the respondent, being exposed to offensive materials, being coerced into an inappropriate social setting, and having sexist comments directed toward the respondent), *unwanted sexual attention* (having a colleague attempt to establish an unreciprocated sexual relationship, and having a colleague attempt to stroke or fondle the respondent) and *sexual coercion* (having a colleague insinuate it was necessary for the respondent to cooperate with sexual advances for professional reasons, experiencing negative consequences for refusing sexual advances, and experiencing positive consequences for accepting sexual advances).

Lastly, the survey asked subjects’ perceptions (from those experiencing unwanted sexual behaviors) of the extent of negative outcomes on their professional self-confidence and career advancement. The scale asked, “Please indicate how much you agree or disagree with the following statements: I have declined a job opportunity or left a job due to my experiences or expectations of perceived SH or gender discrimination; I would elect to pursue a career in anesthesiology if given the option again; I feel anesthesiology is a healthy and positive environment for women; I feel burned out from my work, and I have become more callous toward people since I took this job.” The responses were based on a 1–5 Likert scale, with 1 = strongly disagree; 2 = somewhat disagree; 3 = neutral; 4 = somewhat agree and 5 = strongly agree. The entire survey is available in Appendix A.

### Statistical analysis

Data were collected using Qualtrics (Qualtrics, Provo, UT) survey software and were exported for analysis. Continuous variables were compared between groups using Students’ t-tests and were reported as mean ± standard deviation. Categorical variables were compared between groups using Pearson’s chi-squared tests and reported as frequency and percentage. Hypothesis testing was conducted at a 5% type I error rate. We accepted an alpha of less than 0.05 as statistically significant and all *p*-values were two-tailed. Statistical analyses were conducted using SAS version 9.4 (SAS Institute, Cary, NC).

## Results

### Demographics

**Figure 1:**
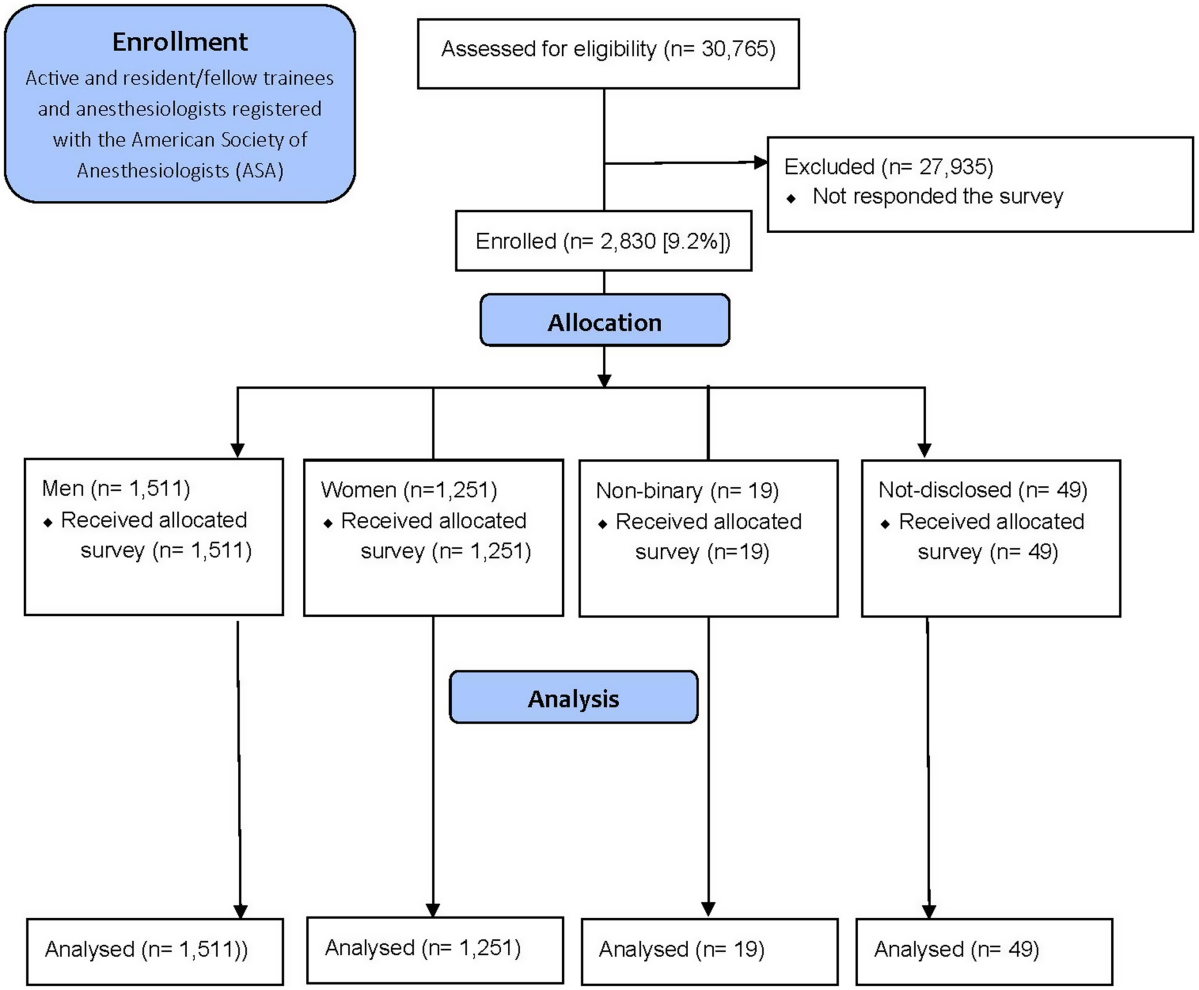
CONSORT 2010 flow diagram – enrollment. N, number; %, percentage.

The email survey was sent to 30,765 registered ASA members, with 5,616 members categorized as resident members (18.3%) and 25,149 (81.7%) active members, representing physician anesthesiologists currently in practice. A total of 2,830 members responded to the 20 items survey for this quantitative analysis, yielding a response rate of 9.2% (Figure 1; Table 1).

**Table 1:** Demographics characteristics.

Variables	All participants (*n* = 2,830)	Men (*n* = 1,511)	Women (*n* = 1,251)	Non-binary (*n* = 19)	Not-disclosed (*n* = 49)
N (%)	2,830 (100%)	1,511 (53.4%)	1,251 (44.2%)	19 (0.7%)	49 (1.7%)
Location of Practice, N (%)					
The United States of America	2,813 (99.4%)	1,501 (99.3%)	1,244 (99.4%)	19 (100%)	49 (100%)
Overseas	17 (0.6%)	10 (0.7%)	7 (0.6%)	0 (0%)	0 (0%)
Position, N (%)					
Trainee (Anesthesiology residents or fellows)	163 (5.8%)	81 (5.4%)	81 (6.5%)	0 (0%)	1 (2.0%)
Attending Anesthesiologists	2,667 (94.2%)	1,430 (94.6%)	1,170 (93.5%)	19 (100%)	48 (98.0%)
Attending Years in Practice					
0–5 years	366 (13.7%)	159 (11.1%)	197 (16.9%)	3 (15.8%)	7 (14.6%)
6–10 years	408 (15.3%)	157 (11.0%)	241 (20.6%)	4 (21.1%)	6 (12.5%)
11–15 years	415 (15.6%)	180 (12.6%)	224 (19.2%)	1 (5.3%)	10 (20.8%)
15+ years	1,474 (55.3%)	931 (65.2%)	507 (43.4%)	11 (57.9%)	25 (52.1%)
Not disclosed	4 (0.1%)	3 (0.2%)	1 (0.1%)	0 (0%)	0 (0%)
Type of fellowship completed					
Pediatric	389 (13.8%)	152 (10.1%)	231 (18.5%)	2 (10.5%)	4 (8.2%)
Cardiothoracic	304 (10.7%)	191 (12.6%)	102 (8.2%)	5 (26.3%)	6 (12.2%)
Pain Medicine	170 (6.0%)	98 (6.5%)	66 (5.3%)	1 (5.3%)	5 (10.2%)
Critical care	166 (5.9%)	103 (6.8%)	57 (4.6%)	2 (10.5%)	4 (8.2%)
Obstetric	130 (4.6%)	57 (3.8%)	72 (5.8%)	0 (0%)	1 (2.0%)
Regional Anesthesiology	107 (3.8%)	48 (3.2%)	56 (4.5%)	1 (5.3%)	2 (4.1%)
Other	138 (4.9%)	70 (4.6%)	65 (5.2%)	1 (5.3%)	2 (4.1%)
None	1,585 (56.0%)	876 (58.0%)	671 (53.6%)	8 (42.1%)	30 (61.2%)

N, number; %, percentage.

Over half of the total respondents (53.4%, *n* = 1,511) identified as men, 44.2% (*n* = 1,251) identified as women, 0.7% (*n* = 19) identified as non-binary and 1.7% (*n* = 49) chose not to disclose their gender. Of these respondents, 99.4% reported practicing in the United States and over half of them (55.3%) had more than 15 years of practice experience. Of the total respondents, 5.8% (*n* = 163) identified as a resident or fellow (trainee) and 94.2% of respondents identified as an attending anesthesiologist. A greater proportion of male respondents (65.2%) had 15+ years of experience compared to female respondents (43.4%; *p*-value< 0.0001).

Lastly, 44% of the attendings completed a fellowship (42% men and 46.4% women, *p*-value0.0145); the most commonly reported fellowships were pediatric (13.8%), cardiothoracic (10.7%), and pain medicine (6%; Table 1).

### Perceived SH occurrences

More than 57% of all respondents perceived some form of SH. A significantly greater proportion of women respondents reported perceived SH compared to men (75.5% women and 44.2% men, *p*-value<0.0001; Table 2). The forms of SH were sub-categorized into gender harassment, unwanted sexual attention, and sexual coercion. The most common forms of *gender harassment* reported were “having sexist comments directed toward the respondent” (15.2% men vs. 58.2% women; *p*-value<0.0001) and “being told sexually suggestive stories” (36.9% men vs. 56.5% women; *p*-value<0.0001). Almost half (45.3%) of all respondents reported being told unwanted sexually suggestive stories (Table 2).

**Table 2:** Type of perceived sexual harassment occurrences.

Variables, N (%)	All participants	Men	Women	*p*-value
N (%)	2,830 (100%)	1,511 (100%)	1,251 (100%)	
Perceived some type of sexual harassment	1,631 (57.6%)	668 (44.2%)	945 (75.5%)	
No to all	1,199 (42.37%)	843 (55.8%)	306 (24.5%)	**<0.0001**
Gender Harassment, N (%)				
Been told sexually suggestive stories	1,282 (45.3%)	558 (36.9%)	707 (56.5%)	**<0.0001**
Had crude sexual remarks directed toward you	629 (22.2%)	196 (13%)	426 (34.1%)	**<0.0001**
Been exposed to offensive display materials (ex., magazines, workroom computer screen savers)	329 (11.6%)	147 (9.7%)	179 (14.3%)	**0.0002**
Been coerced into an inappropriate social setting (ex., attending a strip club)	42 (1.5%)	22 (1.5%)	19 (1.5%)	1.0000
Had sexist comments directed toward you	966 (34.1%)	229 (15.2%)	728 (58.2%)	**<0.0001**
Unwanted Sexual Attention, N (%)				
Had unwanted sexual attention directed toward you	663 (23.4%)	192 (12.7%)	464 (37.1%)	**<0.0001**
Had a colleague attempt to establish a sexual relationship (unreciprocated) with you	218 (7.7%)	71 (4.7%)	145 (11.6%)	**<0.0001**
Had repeated requests for drinks, dinner, etc., despite rejection	175 (6.2%)	47 (3.1%)	128 (10.2%)	**<0.0001**
Sexual Coercion, N (%)				
Had a colleague attempt to stroke or fondle you	275 (9.7%)	80 (5.3%)	191 (15.3%)	**<0.0001**
Had a colleague insinuate it was necessary to cooperate with his/her sexual advances for professional advancement	55 (1.9%)	12 (0.8%)	42 (3.4%)	**<0.0001**
Perceived negative consequences for refusing or rejecting sexual advances	90 (3.2%)	21 (1.4%)	67 (5.4%)	**<0.0001**
Perceived positive consequences for accepting sexual advances	25 (0.9%)	11 (0.7%)	11 (0.9%)	0.6731

N, number; %, percentage; ex., example; etc., etcetera.Bold values are statistical significant p-value.

The rates of *unwanted sexual attention* (12.7% men vs. 37.1% women; *p*-value<0.0001) and *sexual coercion* (5.3% men vs. 15.3% women; *p*-value<0.0001) were also significantly higher in women respondents in comparison to men. The most common form of *sexual coercion* for men and women was “had a colleague attempt to stroke or fondle you” with 9.7% of all participants indicating this perceived SH.

### Perceived sexual harassment context

The surrounding contexts for perceived SH were reported at all levels of training and the results are displayed in Table 3. SH occurs at all career stages, as 20.5% of all respondents perceived SH at least once as a medical student and 45.6% of the 2,667 attending anesthesiologists perceived SH at least once as an attending.

**Table 3:** Perceived sexual harassment context.

Variables, N (%)	All participants	Men	Women	*p*-value
N (%)	2,830 (100%)	1,511 (100%)	1,251 (100%)	
Level of practice when SH occurred at least once				
As an attending anesthesiologist	1,291 (45.6%)	562 (37.2%)	710 (56.8%)	**<0.0001**
As an anesthesiology resident	937 (33.1%)	318 (21%)	605 (48.4%)	**<0.0001**
As a medical student	581 (20.5%)	205 (13.6%)	366 (29.3%)	**<0.0001**
As an anesthesiology fellow	211 (7.5%)	79 (5.2%)	127 (10.2%)	**<0.0001**
Not applicable, I have never been subjected to the experiences	1,067 (37.7%)	775 (51.3%)	249 (19.9%)	**<0.0001**
Setting when SH occurred at least once				
In an operating room or procedure room	1,278 (45.2%)	485 (32.1%)	778 (62.2%)	**<0.0001**
In the hospital and/or clinic other than the operating room or procedure room	1,073 (37.9%)	417 (27.6%)	643 (51.4%)	**<0.0001**
Outside of the hospital or office	315 (11.1%)	137 (9.1%)	173 (13.8%)	**0.0001**
During a one-on-one meeting in a personal office	229 (8.1%)	65 (4.3%)	163 (13%)	**<0.0001**
Mobile phone texts, N (%)	214 (7.6%)	92 (6.1%)	120 (9.6%)	**0.0007**
At a regional or national conference	153 (5.4%)	56 (3.7%)	95 (7.6%)	**<0.0001**
At a division or departmental conference	113 (4%)	29 (1.9%)	82 (6.6%)	**<0.0001**
Other Setting	52 (1.8%)	18 (1.2%)	32 (2.6%)	**0.0093**
Not applicable, I have never been subjected to the experiences	1,151 (40.7%)	825 (54.6%)	279 (22.3%)	**<0.0001**
Perpetrator type				
Colleague of equivalent level of training of a different specialty (ex., General Surgery)	955 (33.7%)	319 (21.1%)	626 (50%)	**<0.0001**
Colleague of equivalent level of training within Anesthesiology	775 (27.4%)	281 (18.6%)	487 (38.9%)	**<0.0001**
Nurse or ancillary staff	665 (23.5%)	433 (28.7%)	222 (17.7%)	**<0.0001**
Patient or patient family member	540 (19.1%)	169 (11.2%)	365 (29.2%)	**<0.0001**
Person in a leadership position directly overseeing your work	538 (19%)	105 (6.9%)	428 (34.2%)	**<0.0001**
Member of the Anesthesia Care Team (CRNA and/or AA)	359 (12.7%)	212 (14%)	141 (11.3%)	**0.0341**
Person in a national leadership role	67 (2.4%)	17 (1.1%)	47 (3.8%)	**<0.0001**
Other	67 (2.4%)	21 (1.4%)	44 (3.5%)	**0.0003**
Not applicable, I have never been subjected to the experiences	1,145 (40.5%)	819 (54.2%)	280 (22.4%)	**<0.0001**
Reaction to the event				
No Reaction	1,135 (40.1%)	537 (35.5%)	579 (46.3%)	**<0.0001**
Anonymously reported the offender to a supervisor	81 (2.9%)	13 (0.9%)	68 (5.4%)	**<0.0001**
Discussed the event with the offender in private following the event	167 (5.9%)	55 (3.6%)	109 (8.7%)	**<0.0001**
Warned other colleagues to be aware of the offender’s behavior	375 (13.3%)	75 (5%)	298 (23.8%)	**<0.0001**
Reported the offender to a governing board (e.g., medical staff office, department, university, licensing board)	136 (4.8%)	21 (1.4%)	114 (9.1%)	**<0.0001**
Discussed the event with the colleague	536 (18.9%)	117 (7.7%)	413 (33%)	**<0.0001**
Other	177 (6.3%)	72 (4.8%)	104 (8.3%)	**0.0002**
Not applicable, I have never been subjected to the experiences	1,140 (40.3%)	815 (53.9%)	279 (22.3%)	**<0.0001**

N, number; %, percentage; SH, sexual harassment; ex., example; CRNA, certified registered nurse anesthetist; AA, anesthesiologist assistant.Bold values are statistical significant p-value.

The setting where the perceived SH event(s) occurred most frequently by both men and women respondents was “in the operating or procedure room” (32.1% vs. 62.2%, *p*-value<0.0001).

Anyone can be a perpetrator as displayed in Table 3. Women reported that their most common perpetrators were colleagues of equivalent training from a different specialty (50%), colleagues of equivalent training from the same specialty (38.9%), and individuals in leadership positions directly overseeing their work (34.2%). In contrast, male respondents reported the most common perpetrators were nurse or ancillary staff (28.7%), colleagues of equivalent training from a different specialty (21.1%) and colleagues of equivalent training from the same specialty (18.6%).

Of the 57.6% of respondents who perceived at least one type of SH, 40.1% of them indicated there was no reaction or reporting to the perceived SH experience. The results suggested a significantly higher incidence rate of no reaction or reporting among women when compared to men (46.3% women vs. 35.5% men, *p*-value<0.0001). The second most common reaction to the perceived SH event(s) among men and women were discussing the event with a colleague (7.7% vs. 33%, *p*-value<0.0001).

### Witnessed SH

As shown in Table 4, approximately 74.1% of respondents indicated that they had witnessed some form of SH in the workplace at least once. More women have witnessed SH than men (80.9% vs. 69.2%; *p*-value<0.0001). The two most common places to witness SH were in the operating room (38.8%) or in a hospital or clinic location (36.4%).

**Table 4:** Witnessed sexual harassment context.

Variables, N (%)	All participants	Men	Women	*p*-value
N (%)	2,830 (100%)	1,511 (100%)	1,251 (100%)	
Witnessed setting when SH occurred at least once				
In an operating room or procedure room	1,099 (38.8%)	479 (31.7%)	604 (48.3%)	**<0.0001**
In the hospital and/or clinic location other than an operating room or procedure room	1,029 (36.4%)	441 (29.2%)	576 (46%)	**<0.0001**
Other setting	433 (15.3%)	272 (18%)	144 (11.5%)	**<0.0001**
Outside of the hospital or office	298 (10.5%)	133 (8.8%)	163 (13%)	**0.0004**
Mobile phone texts	204 (7.2%)	93 (6.2%)	109 (8.7%)	**0.0124**
During a one-on-one meeting in a personal office	134 (4.7%)	51 (3.4%)	83 (6.6%)	**0.0001**
At a regional or national conference	116 (4.1%)	50 (3.3%)	64 (5.1%)	**0.0208**
At a division or departmental conference	101 (3.6%)	39 (2.6%)	61 (4.9%)	**0.0015**
Not applicable, I have never witnessed a colleague subjected to sexual harassment	734 (25.9%)	465 (30.8%)	239 (19.1%)	**<0.0001**
When you witnessed SH				
As an attending anesthesiologist	1,155 (40.8%)	541 (35.8%)	603 (48.2%)	**<0.0001**
As an anesthesiology resident	813 (28.7%)	306 (20.3%)	498 (39.8%)	**<0.0001**
As a medical student	439 (15.5%)	171 (11.3%)	265 (21.2%)	**<0.0001**
As an anesthesiology fellow	164 (5.8%)	63 (4.2%)	99 (7.9%)	**<0.0001**
Not applicable, I have never witnessed a colleague subjected to sexual harassment	1,291 (45.6%)	820 (54.3%)	420 (33.6%)	**<0.0001**
Reaction to the witnessed event				
No reaction/intervention	862 (30.5%)	404 (26.7%)	446 (35.7%)	**<0.0001**
Anonymously reported the offender to a supervisor	136 (4.8%)	63 (4.2%)	71 (5.7%)	0.075
Discussed the event with the offender in private following the event	212 (7.5%)	138 (9.1%)	70 (5.6%)	**0.0005**
Immediately intervened and prevented the event from escalating	264 (9.3%)	124 (8.2%)	138 (11%)	**0.0131**
Warned other colleagues to be aware of the offender’s behavior	541 (19.1%)	185 (12.2%)	350 (28%)	**<0.0001**
Reported the offender to a governing board (e.g., medical staff office, department, university, licensing board)	165 (5.8%)	73 (4.8%)	92 (7.4%)	**0.006**
Not applicable, I have never witnessed a colleague subjected to sexual harassment	1,229 (43.4%)	779 (51.6%)	400 (32%)	**<0.0001**

N, number; %, percentage; SH, sexual harassment; ex., example.Bold values are statistical significant p-value.

The most common response after witnessing a perceived SH event was no-reaction, intervention, or reporting (30.5%), with a significantly higher incidence among women when compared to men (35.7 vs. 26.7%, *p*-value<0.0001).

### Burnout and satisfaction 5-point Likert scale score

**Figure 2:**
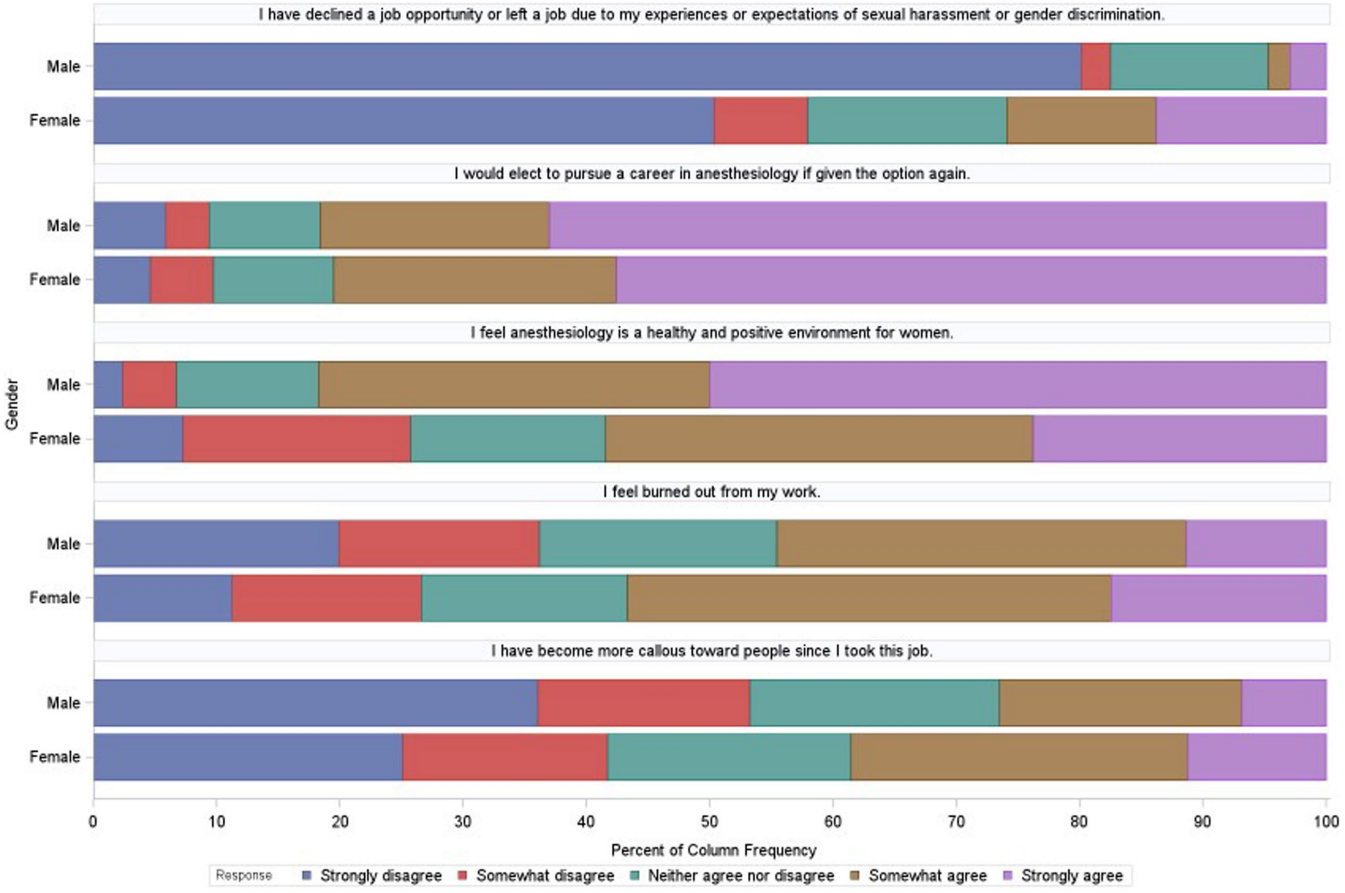
Physician well-being and burnout.

Respondents who perceived SH were more likely to agree with the 5-point Likert-scale questions related to experiencing burnout, facing declining job opportunities or leaving a job, and becoming more emotionally detached from others (Figure 2).

The outcomes of burnout were evaluated using a Chi-squared test, analyzing Likert scale responses to the statement “I feel burned out from my work” among participants who reported experiencing any of the listed SH events within the past 10 years in their professional working environment (*n* = 1,631, 57.6%). Of these, 56.1% (*n* = 915) indicated “somewhat agree” or “strongly agree” to feeling burned out (Table 5). This incidence was higher among women compared to men (60.2% vs. 50.8%, *p*-value< 0.0001). Table 5 presents findings that indicate that respondents who reported experiencing SH were more likely to report feelings of burnout than those who did not report any SH incidents.

**Table 5:** Burnout.

Variables	All participants (*n* = 2,830)	Men (*n* = 1,511)	Women (*n* = 1,251)	Non-binary (*n* = 19)	Not-disclosed (*n* = 49)
N (%)	2,830 (100%)	1,511 (53.4%)	1,251 (44.2%)	19 (0.7%)	49 (1.7%)
Perceived some type of sexual harassment	1,631 (57.6%)	668 (44.2%)	945 (75.5%)		
Answered to “I feel burned out from my work”:					
“Somewhat agree, Strongly agree”	915 (56.1%)	339 (50.8%)	569 (60.2%)		
“Strongly disagree, Somewhat disagree, Neither agree nor disagree”	716 (43.9%)	329 (49.2%)	376 (39.8%)		

N, number; %, percentage; SH, sexual harassment.

## Discussion

The aim of this study was to assess the prevalence of perceived SH reported by members of the ASA. In addition, we sought to assess if there was a relationship between burnout and SH. Over the past several years, there has been increasing attention paid to SH in the workplace and increased research on the detrimental effects it has on women victims’ well-being. According to the United States Equal Employment Opportunity Commission, it is unlawful to harass a person because of the person’s sex or gender (21). Although the law does not prohibit teasing or making offhand comments, SH is illegal (Title VII of the Civil Rights Act of 1964) (12). Experiencing SH perpetuates negative outlooks regarding gender parity in the field (22). These negative effects may be heightened in minorities, gay, lesbian, bisexual, and transgender individuals who often experience SH more frequently (10, 20).

In 2017, Ceppa et al. conducted a survey study of cardiothoracic surgeons, with an 11.82% response rate (6). The study found that 81% of women and 46% of men reported experiencing some form of SH, with a significantly higher incidence among female trainees (90%) compared to male trainees (32%), as well as a strong association between with burnout (6). A multilinear regression model showed that both gender and experienced SH were linked to burnout and an increased trend of declining or leaving one’s workplace (6). Supervising leaders and colleagues were the most common perpetrators for women, while ancillary staff and colleagues were the most common perpetrators for men (6). These findings align with the results of our anonymous, questionnaire-based cross-sectional study, particularly regarding the higher tendency to agree with statements about “burnout feelings” and/or “declining a job opportunity or leaving the workplace” among respondents who reported SH. However, our survey did not account for several other factors contributing to “burnout from work feelings.”

Our survey demonstrates that SH is prevalent in anesthesiology, affecting both trainees and practicing anesthesiologists of all genders. Our data is comparable to the AAMC 2022 report which identified anesthesiology as the specialty with the highest prevalence of SH when compared to other medical specialties. This survey was only administered to ASA members, allowing for a more in-depth analysis of the prevalence of SH in anesthesiology. In 1995, Carr et al. surveyed close to 2,000 academic physicians, finding that 52% of female physicians experienced SH compared to only 5% of male physicians (23). In 2004, Schroen et al. reported that 61% of women surgeons experienced SH and 10% of male surgeons have experienced SH (24). In a 2020 study examining SH, discrimination, and bullying in the orthopedics field, Samora et al. reported that women physicians (81%) are more likely than male physicians (35%) to experience these behaviors (10).

Furthermore, a survey conducted by the International Gynecologic Society in 2018 found that 8 % of respondents reported SH. Among them, 62% felt that the situation was not taken seriously, and 10% felt subject to retaliation by their harasser or superiors (2, 17). Another study examining SH among surgical residents found that 68% of perpetrators were attending surgeons; this may explain why residents may feel apprehensive to report an incident of SH (1).

Previous studies indicate a high incidence of SH but a low incidence of reporting (12). Open discussions of SH and reporting have been associated with a reduction in the incidence of SH and discrimination (12). Therefore, encouraging anonymous reporting is a recommended strategy to prevent retaliation and increase reporting (12). When victims of SH report an incident, they are told that retaliation from the perpetrator is against the law under Title IX (12). However, many victims feel that by reporting an incident it will have negative effects on their career (12, 25). Many studies have highlighted that retaliation is the biggest barrier for reporting SH (12, 25, 26). Furthermore, individuals who are in a minority based on gender, race, ethnicity, or sexual orientation are more likely to experience SH and less likely to report an incident (25, 26).

A study focusing on SH among radiologists found that women in medicine are seven times more likely to experience SH over their male counterparts (2). Another study published the results from a large-scale SH survey that was sent to all faculty and researchers at the University of Michigan Medical School who had been working at the institution for over 1year. This study concluded that over 80% of women experienced SH from individuals within the medical school (27). There are higher rates of SH and abuse from medical institutional insiders than from patients/patients’ families for both men and women. Our study identified colleagues of different or same specialty and nurses or ancillary staff as the most common perpetrators. Perpetrators of SH differed among men and women. Men reported the top two perpetrators were nursing/ancillary staff (28.7%) and colleagues of similar education level in a different specialty (21.1%).

Our results demonstrate similar experiences in anesthesiology. Previous studies in other medical specialties indicate that women physicians experience SH more frequently than men physicians (2, 3, 6, 8). One of the main differences between our study and previous research is that our results indicate a higher incidence of SH among attendings when compared with existing literature in other specialties (2, 3, 17, 28).

## Conclusion

In conclusion, our study highlights the widespread prevalence and impact of SH in the field of anesthesiology, affecting both men and women, with women reporting significantly higher rates than their male counterparts. This ongoing issue presents substantial risks to trainees and anesthesia care providers, contributing to burnout and influencing career choices, such as declining job opportunities or leaving workplaces.

Despite its high prevalence, SH is often underreported and inadequately addressed in training programs and workplaces. These findings underscore the urgent need for comprehensive action plans that include robust policies, mandatory prevention training, and safeguards against SH. Confidential reporting systems and counseling services must be established to support victims, while leadership must foster a cultural shift toward inclusivity, accountability, and harassment-free environments. Regular assessments and targeted interventions are also necessary to effectively address this issue.

Future research should aim to improve sampling strategies, increase response rates, and strengthen the statistical analyses of SH and burnout within anesthesiology. It should also explore the long-term effects of SH on mental health and career progression, as well as evaluate the effectiveness of prevention policies and intervention programs. Additionally, future studies should examine how intersecting factors—such as race, age, LGBTQ+ status, gender identity, and gender expression—contribute to vulnerabilities to SH, offering valuable insights that can be applied to other high-stress medical specialties.

Ultimately, our findings emphasize the pervasive and harmful impact of SH in anesthesiology, disproportionately affecting women. Institutional and cultural reforms are crucial not only to protect individual well-being but also to retain skilled professionals and ensure the quality of patient care.

### Limitations

We acknowledge several limitations of this survey study. First, the voluntary nature of participation may have resulted in a low response rate, particularly due to the sensitive topic of SH. Additionally, the timing of the survey during the COVID-19 pandemic may have further reduced response rates and/or contributed to higher reported burnout levels. Second, the high reported incidence of perceived SH may reflect response bias, with victims more likely to respond, resulting in sampling bias. Some respondents may also have felt uncomfortable answering sensitive questions, which could have impacted the results. Third, the use of the ASA listserv for distribution introduced convenience sampling bias, as not all anesthesiologists are ASA members, limiting generalizability. Fourth, this study also lacked a detailed evaluation of burnout using validated tools like the Maslach Burnout Inventory, which could have provided deeper insights into the relationship between burnout and SH. Lastly, reliance on self-reported data introduces potential response and social desirability biases, and the subjective nature of SH may lead to variability in individual perceptions.

## Data Availability

The raw data supporting the conclusions of this article will be made available by the authors, without undue reservation.
