# Supplementary material for: Perceived sexual harassment and gender differences in anesthesiology: a cross-sectional survey
Source: Front Public Health. 2025 Feb 12;13:1478340. doi: 10.3389/fpubh.2025.1478340 (PMC11864136; doi:10.3389/fpubh.2025.1478340)
Supplement: Supplementary file 1 [file Data_Sheet_1.docx]

1. Do you currently practice Anesthesiology in the United States?

- Yes
- No

1. Please indicate your gender identity

- Male
- Female
- Transgender female (male-to-female)
- Transgender male (female-to-male)
- Genderqueer, neither exclusively male nor female
- Other
- Choose not to disclose

1. Indicate your current number of years in practice

- Currently in training (internship, residency, fellowship)
- 0-5 years
- 6-10 years
- 11-15 years
- 15+ years
- Click to write Choice 6

1. Please indicate if you are currently enrolled in a fellowship in Anesthesiology or which fellowships you have completed (check all that apply)

- Cardiothoracic Anesthesiology
- Obstetric Anesthesiology
- Pediatric Anesthesiology
- Critical Care Medicine
- Regional Anesthesiology
- Pain Medicine
- Other fellowship
- I have not completed a fellowship and/or am not enrolled in a fellowship currently

1. Please indicate whether you have experienced any of the following in a professional environment within the past 10 years (Check all that apply):

- Been told sexually suggestive stories
- Had sexist comments directed toward you
- Had crude sexual remarks directed towards you
- Had unwanted sexual attention directed toward you
- Been exposed to offensive display materials (ex., magazines, workroom computer screen savers)
- Been coerced into an inappropriate social setting (ex., attending a strip club)
- Had a colleague attempt to establish a sexual relationship (unreciprocated) with you
- Had repeated requests for drinks, dinner, etc, despite rejection
- Had a colleague attempt to stroke or fondle you
- Had a colleague insinuate it was necessary to cooperate with his/her sexual advances for professional advancement
- Experienced negative consequences for refusing or rejecting sexual advances
- Experienced positive consequences for accepting sexual advances
- No to all

1. Please indicate your level(s) of practice when you were subjected to at least one of the experiences listed in question #5 (Check all that apply)

- As a medical student
- As an anesthesiology resident
- As an anesthesiology fellow
- As an attending anesthesiologist
- Not applicable, I have never been subjected to the experiences listed in question #5

1. Please indicate the setting in which you were subjected to at least one of the experiences listed in questions #5 (Check all that apply)

- Mobile phone texts
- In the hospital and/or clinic
- During a one-on-one meeting in a personal office
- At a division or departmental conference
- At a regional or national conference
- Outside of the hospital or office
- Other Setting
- Not applicable, I have never been subjected to the experiences listed in question #5

1. Please indicate the perpetrator(s) of the behavior(s) listed in question #5 (Check all that apply)

- Nurse or ancillary staff
- Member of the Anesthesia Care Team (CRNA and/or AA)
- Patient or patient family member
- Colleague of equivalent level of training within Anesthesiology
- Colleague of equivalent level of training of a different specialty (ex., General Surgery)
- Person in a leadership position directly overseeing your work
- Person in a national leadership role
- Other
- Not applicable, I have never been subjected to the experiences listed in question #5

1. Please indicate your reaction to the event(s) (Check all that apply)

No reaction

- Anonymously report the offender to a supervisor
- Discussed the event with the offender in private following the event
- Warned other colleagues to be aware of the offender's behavior
- Reported the offender to a governing body (ex., department, medical staff office
- Discussed the event privately with a colleague
- Other
- Not applicable, I have never been subjected to the experiences listed in question #5

1. Have you witnessed or been made aware of a colleague who has been subjected to any of the following within the past 10 years? (Check all that apply)

- Being told sexually suggestive stories
- Having crude sexual remarks directed toward him/her
- Being exposed to offensive display materials (ex, magazines, workroom computer screen savers)
- Being coerced into an inappropriate social setting (ex., attending a strip club)
- Having sexist comments directed towards him/her
- Having a colleague attempt to establish a sexual relationship (unreciprocated) with him/her
- Having a colleague attempt to stroke or fondle him/her
- Having a colleague insinuate it was necessary for him/her to cooperate to advance professionally
- Experiencing negative consequences for refusing or rejecting sexual advances
- Experiencing positive consequences for accepting sexual advances
- No to all

1. Please indicate when you witnessed a colleague subjected to sexual harassment (Check all that apply)

- As a medical student
- As an anesthesiology resident
- As an anesthesiology fellow
- As an attending anesthesiologist
- Not applicable, I have never witnessed a colleague subjected to sexual harassment

1. What was the gender of the colleague on the receiving end of the sexual harassment?

- Male
- Female
- Both
- Other

1. Please indicate the setting of the witnessed sexual harassment directed at a colleague (Check all that apply)

- Mobile phone texts
- In the hospital and/or clinic
- During a one-on-one meeting in a personal office
- At a division or departmental conference
- At a regional or national conference
- Outside of the hospital or office
- Other setting

1. Please indicate your reaction to the event(s) (Check all that apply)

- No reaction/intervention
- Anonymously reported the offender to a supervisor
- Discussed the event with the offender in private following the event
- Immediately intervened and prevented the event from escalating
- Warned other colleagues to be aware of the offender's behavior
- Reported the offender to a governing board (ex., medical staff office, department)
- Not applicable, I have never witnessed a colleague subjected to sexual harassment

| Please indicate how much you agree or disagree with the following statements: | Strongly disagree | Somewhat disagree | Neither agree nor disagree | Somewhat agree | Strongly agree |
| --- | --- | --- | --- | --- | --- |
| 1. I have declined a job opportunity or left a job due to my experiences or expectations of sexual harassment or gender discrimination. |  |  |  |  |  |
| 1. would elect to pursue a career in anesthesiology if given the option again. |  |  |  |  |  |
| 1. I fell anesthesiology is a healthy and positive environment for women |  |  |  |  |  |
| 1. feel burned out from my work. |  |  |  |  |  |
| 1. I have become more callous toward people since I took this job. |  |  |  |  |  |

1. Please share any specific comments and who made those comments to you that you feel qualify as sexual harassment (answering this question is optional):
